# Regulation of gene expression in roots of the pH-sensitive *Vaccinium corymbosum* and the pH-tolerant *Vaccinium arboreum* in response to near neutral pH stress using RNA-Seq

**DOI:** 10.1186/s12864-017-3967-0

**Published:** 2017-08-07

**Authors:** Miriam Payá-Milans, Gerardo H. Nunez, James W. Olmstead, Timothy A. Rinehart, Margaret Staton

**Affiliations:** 1 Department of Entomology and Plant Pathology, University of Tennessee, Knoxville, TN USA; 2 Horticultural Sciences Department, University of Florida, Gainesville, Florida, USA; 3 Thad Cochran Southern Horticultural Laboratory, USDA-Agricultural Research Service, Poplarville, MS USA; 4 Crop Production and Protection, USDA-Agricultural Research Service, Beltsville, MD USA

**Keywords:** *Vaccinium*, Blueberry, Abiotic stress, pH, RNA-Seq, Differential expression

## Abstract

**Background:**

Blueberries are one of the few horticultural crops adapted to grow in acidic soils. Neutral to basic soil pH is detrimental to all commonly cultivated blueberry species, including *Vaccinium corymbosum* (VC). In contrast, the wild species *V. arboreum* (VA) is able to tolerate a wider range of soil pH. To assess the molecular mechanisms involved in near neutral pH stress response, plants from pH-sensitive VC (tetraploid) and pH-tolerant VA (diploid) were grown at near neutral pH 6.5 and at the preferred pH of 4.5.

**Results:**

Transcriptome sequencing of root RNA was performed for 4 biological replications per species x pH level interaction, for a total of 16 samples. Reads were mapped to the reference genome from diploid *V. corymbosum*, transforming ~55% of the reads to gene counts. A quasi-likelihood F test identified differential expression due to pH stress in 337 and 4867 genes in VA and VC, respectively. Both species shared regulation of genes involved in nutrient homeostasis and cell wall metabolism. VA and VC exhibited differential regulation of signaling pathways related to abiotic/biotic stress, cellulose and lignin biosynthesis, and nutrient uptake.

**Conclusions:**

The specific responses in VA likely facilitate tolerance to higher soil pH. In contrast, response in VC, despite affecting a greater number of genes, is not effective overcoming the stress induced by pH. Further inspection of those genes with differential expression that are specific in VA may provide insight on the mechanisms towards tolerance.

**Electronic supplementary material:**

The online version of this article (doi:10.1186/s12864-017-3967-0) contains supplementary material, which is available to authorized users.

## Background

*Vaccinium* is an economically important genus within the Ericaceae family that includes blueberries (*Vaccinium* spp. subgenus *Vaccinium*) and cranberries (*Vaccinium* spp. subgenus *Oxycoccus*). *V. corymbosum* (highbush blueberry, VC) is a high-value fruit crop that exhibits strict soil adaptation to acidic soils with high organic matter content [1–3]. These characteristics are relatively uncommon in agricultural soils, and when grown in higher pH soils, highbush blueberries exhibit nutrient deficiencies, stunted growth, and reduced yield [4]. Thus, growers have to use soil amendments and fertigation to provide adequate conditions for cultivation [5, 6].

*V. arboreum* (sparkleberry, VA) is a wild blueberry species native to the southeastern U.S. [7, 8] that, according to field observations, exhibits wider soil adaptation than VC. Previous research indicates that VA can tolerate soil pH above 6.0 [7] and grows in soils that would normally exclude other blueberry species [7]. Additionally, VA exhibits greater ability to take up nitrate –the most abundant form of nitrogen at higher pH– and iron –an element with reduced solubility at higher pH– than VC [9]. This has motivated the use of VA as a rootstock for blueberry production [10] and a genetic source for blueberry breeding [11]. Yet, few aspects of the soil adaptation of VA have been studied to date. Neither VC nor VA are capable of acidifying the rhizosphere directly [12]. Other mechanisms of tolerance have yet to be proposed and tested.

Soil acidity is a major constraint for most cultivated plants worldwide [13]. However, most blueberries stand out given their adaptation to grow in acidic soils and inhibition at pH above 6.0, making them calcifuge plants [9]. The effect of acidity on root health and growth, derived from H^+^ ion excess, depends widely on plant species [13]. Furthermore, in non-calcifuge plants, regulation of the concentrations of nutrients and aluminum in the solution improves tolerance to H^+^ ion in several species [13]. At the molecular level, plant responses to acidic soils can be broadly categorized into two groups: responses to the high concentration of H^+^ ions in the soil matrix and responses to nutrient availability at acidic soil pH.

High concentrations of H^+^ ions have been shown to affect cell wall and plasma membrane integrity and functionality in susceptible plants. For instance, low pH stress weakens Ca^2+^-mediated cross-linkage of pectin, which destabilizes primary cell walls [14]. In response, the expression of genes whose products are involved in cell wall reinforcement, such as pectin esterases and arabinogalactan proteins is upregulated [15]. Exposure to low pH also leads to plasma membrane depolarization [16], reduction of proton extrusion by plasma membrane H^+^-ATPases [17], and enhancement of the relocation of excess H^+^ from the cytosol to the vacuole through vacuolar V-H^+^-ATPase and V-H^+^-pyrophosphatase pumps [18]. Other means of cytosolic pH homeostasis, such as glutamate decarboxylation [19], might also be involved. Furthermore, acidification of the cytoplasm of plants grown in acidic conditions leads to accumulation of reactive oxygen species (ROS) and subsequent activation of antioxidative mechanisms in the roots [20, 21]. Oxidative damage is likely to be responsible for the damage to root epidermal cells observed in non-calcifuge plants grown in acidic soils and media [22].

The second group of plant responses to soil acidity is related to responses to pH-dependent changes in nutrient bioavailability. Al, Fe, Mn, Cu, Zn, and B are more bioavailable at acidic than at neutral soil pH. Thus, soil acidity facilitates plant uptake of these nutrients [23, 24]. However, soil acidity dissolves toxic levels of aluminum in the soil matrix, which impairs plant growth and is a major limitation for agricultural production in acidic soils [25, 26]. On the other hand, P, K, Mg, Ca and Mo become less bioavailable as soil pH decreases. Soil acidity limits plant uptake of these nutrients [24].

Blueberries are some of the most economically important calcifuge plants and serve as a horticultural model for study of acidic soil adaptation. However, the development and adoption of genomic tools to study blueberry have been slow. In the wild, blueberries range in ploidy level from diploid to hexaploid [27]. Cultivated *V. corymbosum* hybrids are autotetraploid [28], which has limited their study as most genomic tools are developed for diploid organisms. The first transcriptome in blueberry was introduced in 2012 and it consisted of approximately 15,000 contigs [29]. This was followed by a genetic linkage map (for diploid blueberry) composed of 12 linkage groups (equivalent to the haploid chromosome number) covering 1740 cM [30]. Later, the first draft genome assembly was constructed using HiSeq Illumina reads [31]. The annotation of this genome provided a nonredundant set of 70,581 gene models representing 63,840 genes [32], from which 51,515 genes were annotated with a protein sequence of at least 30 residues. Recently, the first genetic map in autotetraploid blueberry was produced using SNP markers generated through genotyping by sequencing and linkage-mapping software specialized for autotetraploids [33]. Altogether, these developments have made it possible to apply omics technologies to the study of blueberries and their relatives.

The present study used transcriptomic tools to examine VC and VA responses to growth at two different nutrient solution pH levels. While the vast majority of research in the topic of plant responses to soil pH has been carried out on non-calcifuge species, here we present a study comparing the transcriptomes of two congeneric plant species with contrasting soil adaptations; VA exhibits tolerance to higher pH soil while VC is inhibited. We hypothesized that genes that are differentially expressed between pH 4.5 and pH 6.5 in VC and VA are related to the contrasting soil adaptation of these two species. To test this, we conducted greenhouse hydroponic experiments with four biological replicates of each condition in each species and profiled the root tissues with high throughput transcriptome sequencing (RNA-Seq).

## Methods

### Biological material

To facilitate the application of differential pH treatments, we utilized a hydroponic growth system described previously [9, 12]. The hydroponic nutrient solution contained 0.5 mM KNO_3_, 0.5 mM K_2_HPO_4_, 1.0 mM MgSO_4_, 0.5 mM CaCl_2_, 0.045 mM H_3_BO_3_, 0.01 mM MnSO_4_, 0.01 mM ZnSO_4_, with 0.3 μM CuSO_4_, and 0.2 μM Na_2_MoO_4_. The nutrient solution was buffered at the appropriate treatment pH with 5.0 mM 2-(N-morpholino) ethanesulfonic acid (MES) and an iron concentration of 45 μM was provided using Sequestrene 330 (10% iron (III)-diethylenetriamine pentaacetic acid) (Becker Underwood, Inc.). The nutrient solution was contained in continuously aerated 2 L bottles wrapped with foil covering (see Additional file 1: Figure S1). Nutrient solution was changed weekly. All plants were maintained in a greenhouse that ranged between 10 °C to 35 °C with a photosynthetic photon flux averaging approximately 425 μmol·m^−2^·s^−1^.

Approximately 1-year-old rooted cuttings for VC cv. ‘Emerald’ and VA clonal selection FL09–502 were removed from their pots, the peat media washed from the roots, and individually transplanted to the 2-L vessels containing the nutrient solution on 15 April 2010. The plants were allowed to equilibrate in the hydroponic nutrient solution maintained at pH 5.5 until 3 May 2010 when pH treatments were applied to the VC and VA plants. Twenty plants each of VC and VA were organized in a completely randomized pattern and the differential pH treatments (4.5 and 6.5) were applied, with 10 plants of each species receiving a pH treatment. Of the 10 plants per species in each pH treatment, five were randomly selected to be used for destructive ferric chelate reductase (FCR) root enzymatic activity assays. FCR activity has previously been shown to differ between VC and VA [9, 34], and VC varieties may show iron deficiency because of reduced FCR activity [35]. Differential FCR activity in the roots and leaves was used as a proxy indicator of the potential differential gene expression between VC and VA.

### RNA isolation

At the conclusion of treatments, whole plants in nutrient solution were moved immediately to the lab and the root systems were harvested for total RNA isolation. Plants were removed from the nutrient solution vessels, roots were briefly rinsed in deionized water, blotted dry, separated from the main plant and weighed. Root systems for each plant were separated into multiple groups containing a main root arising from the crown and all the subtending fine roots and immediately frozen in liquid nitrogen. Root samples were stored in a − 80 °C freezer until RNA isolation. Total RNA was isolated from the root samples using an RNAqueous total RNA isolation kit (ThermoFisher Scientific).

### Illumina sequencing library construction

Total RNA was quantitated on a NanoDrop Spectrophotometer (NanoDrop Technologies, Inc.), and sample quality was assessed using the Agilent 2100 Bioanalyzer (Agilent Technologies, Inc). Following this, rRNA was removed from 1.4 μg aliquots of total RNA using the Ribo-Zero™ rRNA Removal Kit for plant tissues (Epicentre) following the manufacturer’s protocol. Then, rRNA-depleted RNA was used for library construction with the ScriptSeq v2 RNA-Seq library preparation kit (Epicentre) according to manufacturer’s protocol. Briefly, rRNA-depleted RNA was fragmented by incubation with divalent cations at 85 °C, followed by reverse transcription using random primers containing a 5′-tagging sequence. 3′-tag was added by the terminal-tagging reaction as a result of di-tagged, single-stranded cDNA. Following purification, the di-tagged cDNA was amplified by limited-cycle PCR, which incorporated the Illumina adapter sequences. Amplified libraries were purified by Agencourt AMPure beads (Beckman Coulter). Library size and mass were assessed in the Agilent 2100 Bioanalyzer. A 200–2000 broad library peak was observed with the highest peak at ~500 bp. Quantitative PCR (qPCR) was used to validate the library’s functionality using the KAPA library quantification kit (Kapa Biosystems). Finally, libraries were pooled in equimolar concentration and submitted for paired-end 100 bp sequencing in the Illumina HiSeq 2000. RNA-Seq library construction and sequencing were performed at the University of Florida Interdisciplinary Center for Biotechnology Research.

### Methods of read mapping and transcriptome analysis

Reads produced by the Illumina HiSeq system were cleaned up with the Trimmomatic program version 0.35 [36]. Sequencing adapters were trimmed off and low quality (average quality <15) and short reads (<30 bases) were excluded from RNA-Seq analysis. Read quality control was performed using FastQC [37]. The resulting fragments were mapped to a blueberry draft genome [31] using GSNAP version 2016–04-04 [38] allowing a maximum of 10% mismatches in each read. The algorithm in GSNAP allows detection of complex variants involving multiple mismatches, long indels, and splicing in short reads, accepting user-provided databases of known exon–intron boundaries. This approach prevents false positive and negative results from probabilistic models. Mapping quality control metrics were obtained with RNA-SeQC v1.1.8 [39]. Read counts were obtained using the standalone script provided by HTSeq [40]. The final expression profile was represented by a matrix of read counts, with one row for each gene model and one column per sample.

### Analysis of differential gene expression

Digital gene expression was analyzed with edgeR [41]. edgeR contains statistical methodology based on the negative binomial distribution and is suited for analysis of genomic data that produce counts, such as RNA-Seq. Genes with less than one count for each million mapped reads (cpm) in at least four samples -the number of replicates per species and treatment- were filtered out. For this study, a multifactorial experimental design was parametrized with four groups, with the species the major group and each pH condition a sub-group. Differential expression (DE) was tested by performing a quasi-likelihood F-test, which provides robust and reliable error rate control when the number of replicates is small [42]. Significant up- and down-regulated genes were identified after performing multiplicity correction by applying the Benjamini-Hochberg method on the *p*-values to control the false discovery rate (FDR). Less than 0.5% of the DE genes showed no expression in one of the species.

### Annotation of reference gene models

The reference transcript models were re-annotated as follows. First, new encoded peptides were predicted using TransDecoder 3.0.0 [43]. All open reading frames (ORFs) that were at least 50 amino acids long were identified within the models. These ORFs were scanned for homology to known proteins using blastp [44] against the UniProtKB/TrEMBL [45] database for plants, and searched against Pfam [46] for conserved protein domains with HMMER [47]. TransDecoder was rerun, using the outputs generated from blastp and hmmer, to improve coding region selection.

Functional gene annotations were assigned based on sequence similarity of reference transcripts to proteins in the UniProtKB/TrEMBL database for plants. To avoid redundancy in annotation results, the isoform with the best match was selected for each gene. In the absence of a match, the longest isoform was selected to represent the gene. Finally, the nonredundant sequence sets were further annotated blasting the transcripts against RefSeq proteins for *Arabidopsis thaliana* and using InterProScan 5.15–54.0 software [48] for assignment of GO terms (Gene Ontology Consortium). Further annotation in *Arabidopsis* was retrieved using the R package org.At.tair.db [49].

### Gene ontology enrichment analysis

AgriGO version 1.2 [50], a web-based analysis toolkit, was used to perform singular enrichment analysis (SEA) of GO terms in the lists of DE genes for VA and VC. The transcriptome GO term annotations from InterProScan were formatted following the specifications of the tool and used as customized annotation of background reference. Calculation used the hypergeometric statistical test method, the Yekutieli multi-test adjustment method for FDR correction under dependency, and a significance level of 0.05. Complete GO was selected as Gene Ontology type. REVIGO [51] was used to summarize and visualize results.

## Results

### Phenotypic difference of VA and VC under different pH treatment

**Fig. 1:**
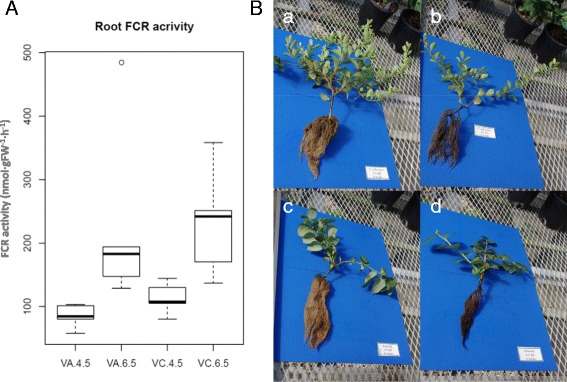
Root FCR activity (**a**) and appearance of blueberry plants (**b**) after acclimation to pH 4.5 or 6.5. Harvest of plants proceeded upon exhibition of differential phenotype dependent on pH treatment. **a** Values for ferric chelate reductase (FCR) activity were significantly different on each blueberry species after 8 weeks of treatment (*P* < 0.05). *n* = 5. **b** Root health had diverged in plants from VA (a, b) and VC (c, d) after 6 weeks of treatment at pH 4.5 (a, c) or 6.5 (b, d)

After 60 days in differential pH treatments, the experiment-wide root FCR activity between the pH 4.5 (99.4 nmol·gFW^−1^·h^−1^) and 6.5 (229.7 nmol·gFW^−1^·h^−1^) treatments was significantly different (*P* < 0.004). Significant change (*P* < 0.05) was also depicted by each species (Fig. 1a). Using this as an indication of potential root level responses to pH treatment, the experiment was concluded and root tissue harvested for total RNA isolation. The appearance of the roots at the end of the treatments showed a much healthier state at pH 4.5, whereas those grown at pH 6.5 had blackened (Fig. 1b).

### Mapping efficiency of RNA-Seq reads to the blueberry reference genome

**Fig. 2:**
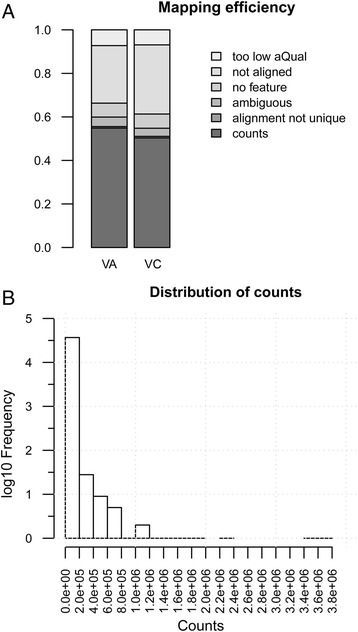
Quality metrics of read mapping performance (**a**) and density plot of counts per gene model (**b**)

RNA-Seq reads from two blueberry species, 207.7 million from diploid VA and 452.4 million from tetraploid VC were mapped to a reference draft genome generated for a diploid *V. corymbosum* [31]. The utilization of a reference genome from a close relative provides a predefined gene namespace that facilitates differential expression analysis [52]. Also, mapping paired-end RNA-Seq data to reference sequences may recover expression data for a higher number of genes compared to mapping to a de novo assembly [53]. On average, 67% of the initial reads were uniquely mapped to the reference genome using GSNAP. Average mapping rate of reads did not show significant differences in relation to the species analyzed (*p* < 0.05, student’s *t*-test). GSNAP detected ~5–7% spliced reads (i.e. reads spanning an intron/exon boundary). In both species, ~85–90% of the mapped reads mapped within a gene, however, only ~70% map exclusively to annotated exons. This may indicate a need for further refinement of the gene models for the reference genome. As genome annotation is beyond the scope of this work, only reads that mapped uniquely and with high quality to annotated gene exons were used to assess gene expression. These informative reads accounted for 55% and 50% of total reads in VA and VC, respectively (Fig. 2a). Overall, this method detected 33,320 and 40,317 genes in VA and VC, respectively.

### Overrepresented genes in blueberry samples

Approximately, 91% of gene models had less than 10,000 counts per gene model. A few genes with extremely high counts were observed (Fig. 2b), encoding subunits of the mitochondrial membrane respiratory chain complexes NADH dehydrogenase (complex I; NADH-ubiquinone oxidoreductase chains 4 and 5), cytochrome b-c1 (complex III; cytochrome b) and ATPase (a CF_0_ subunit). Requirements for ATP in actively growing tissues such as root is high, increasing the rate of respiration to supply the demand of energy [54]. High expression of a sucrose synthase also highlights sugar metabolism in roots, which are the major sink organs for photosynthetic derived sugars, producing UDP-glucose for cell wall and glycoprotein biosynthesis. Energy production in roots depends on mitochondrial respiration and glycolysis, which are enhanced under certain biotic or abiotic stresses such as osmotic stress, pathogenesis and flooding [55, 56].

### Evaluation of sample similarity in relation to species and pH treatment

**Fig. 3:**
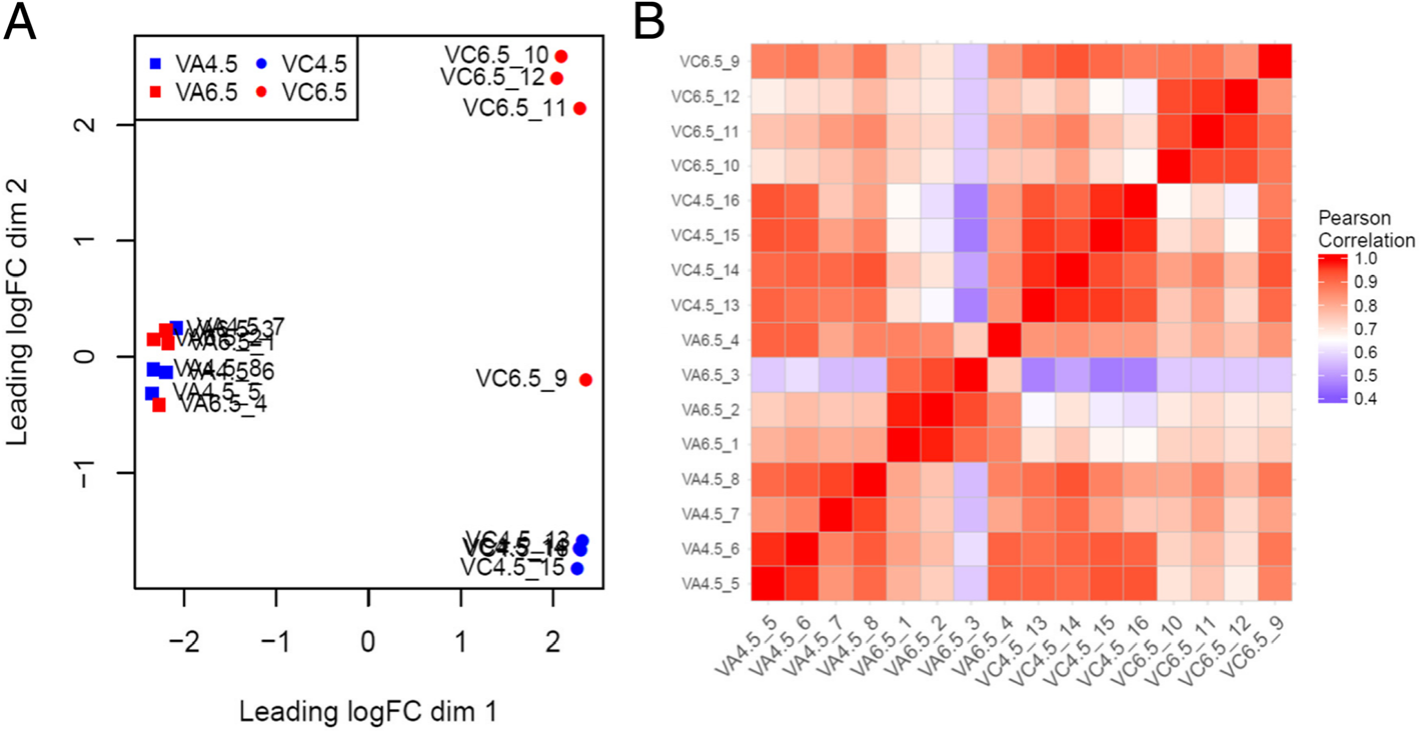
Representation of distances between samples as MDS plot of leading log-fold-change (**a**) and heatmap of sample-sample correlation distances (**b**)

Multidimensional scaling (MDS) was used to separate samples in 2 dimensions by leading log-fold-change (logFC), i.e. the root mean square average of the largest log2-fold-changes between each pair of samples (Fig. 3a). The first dimension clearly separated each species, while the second dimension aggregated most samples of VC by pH. This clear difference in gene expression patterns between preferred and unpreferred soil pH is expected in VC, the calcifuge species. Interestingly, all samples of the higher pH-tolerant VA clustered together, showing much less gene expression change in response to pH. This suggests a root system not significantly stressed by higher pH soil or very efficient at combatting soil pH stress and thus not responding with major shifts in gene expression. Global similarity was further explored through a correlation matrix of samples (Fig. 3b). Most of the samples intercorrelated within blocks responding to the combination of species and pH treatment. Gene expression of stressed plants was also species-specific in contrast to the high correlation among species present in plants grown at optimal pH. Plants grown under stress showed less overall correlation to each other than plants grown in control conditions. Interestingly, samples 4 (VA at pH 6.5) and 9 (VC at pH 6.5) showed slightly higher correlation to non-stressed plants compared to other plants in their respective groups.

### Differentially expressed genes on VA and VC responsive to pH change

When mapping to a closely related reference genome, most genes are expected to have remaining high sequence similarity, thus enabling quantification. The high level of genomic conservation is further confirmed by the ability to produce fertile hybrids by crossing VC to VA seedlings that have undergone chromosome doubling [11, 57]. However, some genes may have recently diverged in sequence or been deleted from the genome. To avoid these genes in downstream analysis, genes with very low counts were removed, leaving 36,891 genes for differential expression (DE) analysis. The performance of the initial steps of filtering and normalization of the count data is represented on a MA-plot (see Additional file 2: Figure S2).

**Fig. 4:**
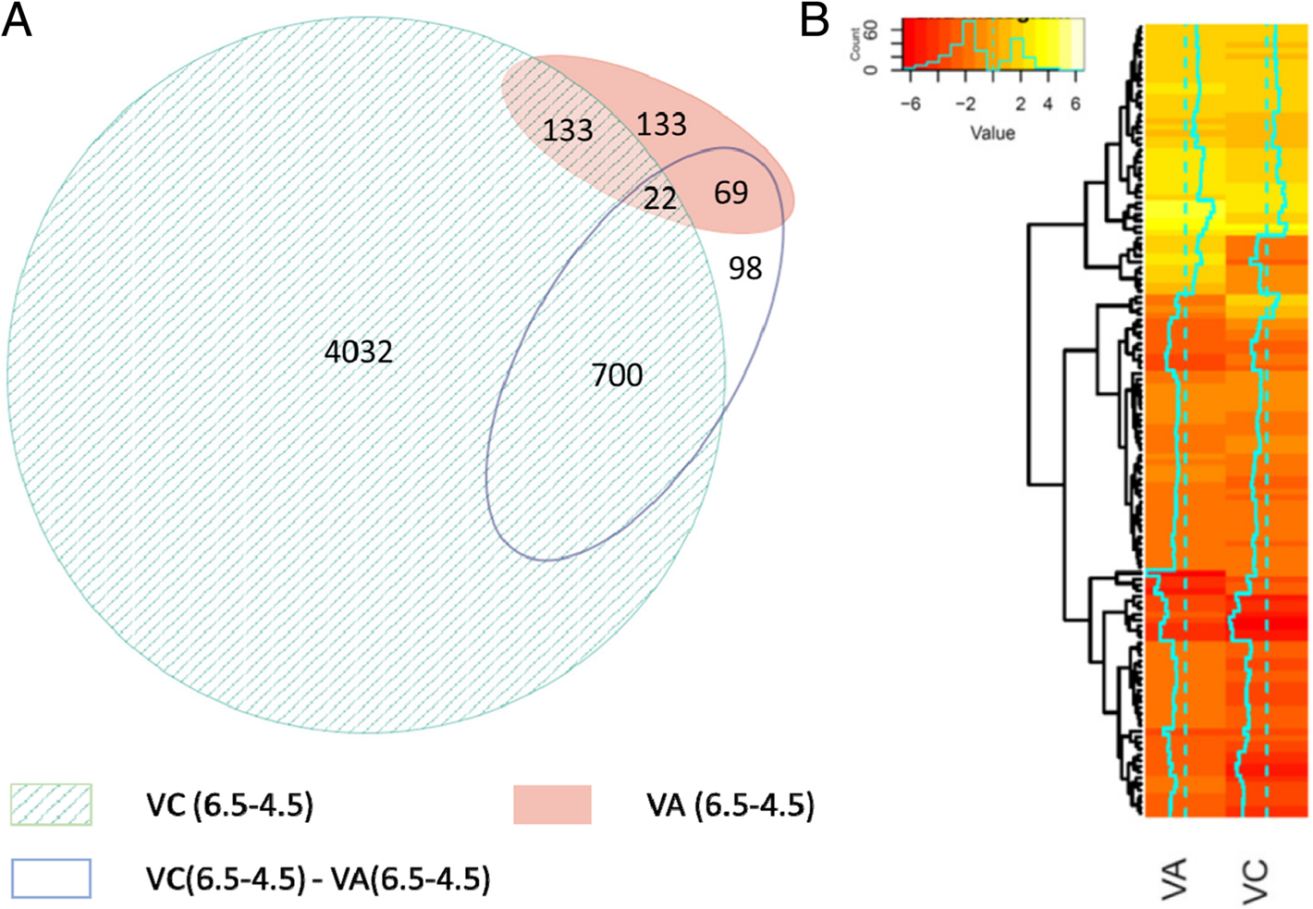
Venn-diagram (**a**) and heatmap (**b**) of genes with differential expression under pH treatment. Differential expression was assessed using quasi-likelihood F-test. Four coefficients were determined using each species (*Vaccinium arboreum* and *V. corymbosum*) as a group and each pH condition (4.5 and 6.5) as sub-group. **a** The contrasts show genes responding to higher pH in each species (*red circle* for VA and striped circle for VC) and those responding differently between species (empty *circle*). The figure was produced using eulerAPE [89]. **b** Heatmap representation of fold change of the overlapping subset of differentially expressed genes between VA and VC

Customized contrasts testing for differential expression were achieved after fitting a linear model based on the quasi-likelihood method and using a design matrix with four groups, representing the combinations of species and pH treatment. Less than 0.5% of reported genes had no representation in one of the species, indicating that almost all DE genes are detectable by read mapping from both species using the diploid reference genome. The first tested contrast, for response to pH treatment in VC, yielded 4867 DE genes (Fig. 4a; striped *circle*). A second contrast, of pH treatment in VA, yielded 337 DE genes (Fig. 4a; *red circle*). A final test was conducted to see how the two species respond differently to the increased pH treatment. This contrast revealed 889 genes that respond differently at higher pH in VC in relation to VA response (Fig. 4a; empty *circle*). The ratio of upregulated/downregulated genes in each of the three contrasts were 1852/3015, 178/159, and 291/598, respectively (see Additional file 3: Table S1 for the lists of genes).

### Functional annotation of DE genes

Custom gene annotations were prepared using the previously published 70,581 gene models representing 63,840 genes [32]. We predicted encoded peptides from the transcript models using knowledge of homology results for frame selection and established functional annotations based on homology to known proteins (Additional file 4: Table S2). For the open reading frame identification, putative protein sequences at least 50 amino acids long were selected from the transcript models, obtaining 50,456 peptides. The best candidate peptide sequence was retained for each gene, resulting in 44,332 peptides. Functional annotation was provided by homology searching against the plant TrEMBL protein database, *Arabidopsis thaliana* proteins, and the InterPro protein signature databases. Blast searching identified hits for 58% of the gene models at a 0.001 evalue, from which 13% indicated “Uncharacterized protein” (Additional file 4: Table S2). InterPro annotation of proteins assigned GO terms for 44,731 genes.

**Fig. 5:**
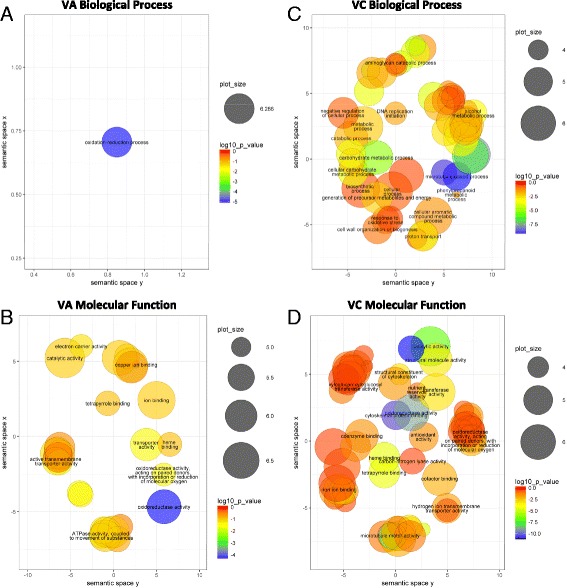
Plot of significant enriched GO terms in *V. arboreum* and *V. corymbosum* under pH stress. Enrichment was estimated for genes showing differential expression in *V. arboreum* (**a**, **b**) and *V. corymbosum* (**c**, **d**) in response to treatment pH 6.5 compared to control pH 4.5. Biological process (**a**, **c**) and molecular function (**b**, **d**) ontology terms were analyzed independently on AgriGO and terms below 0.05 FDR after Yekutieli multi-test adjustment were plotted using REVIGO as function of namespace. Each circle represents a significant GO term, with semantically similar terms placed closer together. Circle color indicates the *p*-value obtained from AgriGO and size of the circle corresponds to the occurrence of the term in the underlying data set (i.e. more general terms appear as larger *circles*). A single representative GO term label is provided for each cluster for clarity; this term is selected by most significant *p*-value. All terms can be found in Additional file 5: Table S3

These GO terms were used to identify enriched biological and molecular processes represented in the DE genes from both VA and VC using the blueberry reference gene models as background (Fig. 5). We used REVIGO to produce a simplified plot for fast visualization of overall functional categories by grouping terms by semantic similarity; the full lists of analyzed terms are provided in Additional file 5: Table S3. In agreement with the different numbers of genes that were DE in VA and VC, the amount of enriched terms is much lower in VA compared to VC (left vs right panels in Fig. 5).

The single process observed in VA (Fig. 5a), oxidation reduction, is also highly significant in VC (Additional file 5: Table S3B). Among the multiple genes annotated with this term, there are several laccases in both species, which are multi-copper binding enzymes involved in lignin catabolic process. Interestingly, while both blueberries show enrichment in copper ion binding function, this process is more significant in VA compared to other ion binding functions, according to FDR, than VC. A differential response that involves control of cell water content is found in transporter activity, in which VA is represented by aquaporins while VC is not enriched in those genes. The differences in functional enrichment between VA and VC target putative candidates in the process towards tolerance to higher pH in VA.

VC shows enrichment in proteins involved in the modification of different components of the cell wall, including xyloglucan endotransglucosylase/hydrolases and cellulose synthases that modify cell wall polysaccharides, and laccases, which participate in lignin biosynthesis. In addition, very diverse significantly enriched functional groups are observed, e.g. those formed by proteins participating in glycolysis, motor proteins like kinesins, ribosomal proteins, helicases for DNA replication and nutrient proteins like cupins and germins. The complexity of terms included in biological process and molecular function are indicative of mechanisms that are triggered in roots in response to the damage caused by the higher pH (Additional file 5: Table S3B). Some of the enriched processes involve intracellular transport (microtubule-based movement), cell wall metabolism, energy (ATP synthesis) and substance metabolism (carbohydrate and fatty acid biosynthesis). In contrast to VA, VC is enriched in response to oxidative stress by transcriptional regulation of multiple peroxidases, supporting the increased susceptibility to pH stress. The implications of these observations will be further discussed in the following sections.

## Discussion

### Digital gene expression from RNA-Seq data

Previous studies identified various responses to low soil pH in higher plants [13]. However, the use of herbaceous species [14, 22] and short induction times [15, 58] likely limited the range of responses to low pH stress that could be documented in those experiments. In fact, some authors have referred to these responses as low pH shock, as opposed to low pH stress responses [18]. In this study, plants were acclimated to high or low pH conditions for over 8 weeks before RNA isolation and transcriptome analysis. This was done with the intention of surveying the plant responses to pH stress, and not pH shock. Additionally, in contrast with the existing literature [14, 15, 22], this study focused on the response to higher pH stress in calcifuge species. To the best of our knowledge, this is the first time this response has been studied using genomic tools. Furthermore, the use of hydroponic cultivation system allowed a strict control of the root environment. Even though the analysis contrasted responses due to pH, we acknowledge that growth in synthetic media such as hydroponic system likely has an effect in the observed results that may be further investigated.

Non-model species usually present some challenges to the use of high-throughput sequencing technologies. Counting expression levels from cell RNA requires a reference genome (or transcriptome) for mapping reads in order to obtain quantitative data. High quality references are available for an increasing number of eukaryotic species in public databases. As examples, there are 99, 235 and 77 reference genomes at NCBI completed at least to the level of chromosome in animals, fungi and plants, respectively (August 2016). However, when considering all levels of assemblies, plants are left far behind, with 919, 1717 and 278, respectively. Furthermore, polyploidy is a common event in plants, posing some extra challenges. In relation to high-throughput technology for differential gene expression, RNA-Seq transcriptome sequencing is a reliable methodology, showing good overall agreement with the traditional method of real-time qPCR [59, 60]. In the present study, RNA-Seq data was generated from diploid VA and tetraploid VC blueberries and mapped to the draft genome generated from diploid *V. corymbosum*. Our mapping strategy used the GSNAP tool to account for spliced reads and allow up to 10% mismatches, obtaining ~67% mapping rate. Customization of tool parameters improved the performance compared to a similar study that mapped 47% baboon reads to a human reference with default options of GSNAP [61]. Both in blueberry and baboon mapping strategies, around 88% of the mapped reads aligned to an intragenic feature.

Gene models generated with computational methods without further manual annotation carry some challenges, particularly the occurrence of split, joined and incomplete genes as well as the lack of discovery of all isoforms. Thus, count data was obtained for genes instead of individual transcripts. Read alignments were processed with HTSeq, obtaining 50–55% effective counts after filtering of low quality or informative reads. Considering the results, the proportion of reads that are finally transformed into counts respond mainly to (i) the mapper program; (ii) the quality of the reference genome; and (iii) the genetic divergence between organisms. The resulting gene profiles showed good correlation within sample groups, suggesting that the decrease observed from mapped reads to final counts was not biased. The distribution observed on the MDS plot separated species and effect of pH treatment on the first two dimensions, which correlated well to pH stress resistance and sensitivity depicted by VA and VC, respectively.

Samples were grouped by species and pH treatment in order to perform multiple contrasts and obtain differential expression in the edgeR package. The main questions addressed were how VC responds to pH stress [VC (6.5–4.5)], how VA responds to pH stress [VA (6.5–4.5)] and what genes are driving the differences in response to stress between the species [VC (6.5–4.5) - VA(6.5–4.5)]. Despite VC having 14 times more genes responding to pH stress, they only overlapped with 40% of the genes regulated by VA, most of which were regulated in the same direction (Fig. 4b). In view of the results, pH tolerance in VA is effective upon regulation of a small subset of key genes compared to the wide effects observed in VC probably due to chemical damage. A list of the most significant differentially expressed genes affected by pH on each species and their differential response is shown in Table 1; some of them were mentioned in the results of GO enrichment, including laccases, cupins and ribosomal proteins. Pathways affected by pH stress will be further discussed in the following sections (a summary of the discussed genes is available on Additional file 6: Table S4).

**Table 1:** Top differentially expressed genes responding to near-neutral pH stress in susceptible and tolerant blueberries

gene_id	logFC	logCPM	*P* value	FDR	uniprot_id	Evalue	Description
A. Vaccinium corymbosum							
CUFF.22809	−5.07	4.814549	1.80E-11	6.63E-07	A0A118JUM8_CYNCS	8.00E-96	Cupin 1
CUFF.4259	−4.52	5.452318	1.37E-10	2.52E-06	A0A103XEL9_CYNCS	1.00E-28	Cupredoxin
CUFF.236	−4.30	4.41962	6.74E-10	5.61E-06	A0A061DQL9_THECC	4.00E-37	Ralf-like 33, putative
CUFF.28304	−3.62	2.988055	9.58E-10	5.61E-06	A0A0B4VRZ0_SALMI	0	Cytochrome P450 CYP86A91
CUFF.40209	−3.79	4.908315	9.60E-10	5.61E-06	B9TAC6_RICCO	0	Receptor protein kinase CLAVATA1, putative
CUFF.54833	−3.94	1.964193	1.22E-09	5.61E-06	B9I5Q8_POPTR	0	Clavata1 receptor kinase family protein
CUFF.8465	−3.9	4.05831	4.77E-10	5.61E-06	I7AUB2_9ROSI	0	Hydroxycinnamoyl coa shikimate/quinate hydroxycinnamoyl transferase (HCT)
CUFF.1958	−6.02	6.395831	1.85E-09	7.60E-06	A0A061GZH4_THECC	3.00E-146	Late embryogenesis abundant (LEA) protein-related
CUFF.38453	−3.16	3.680711	2.07E-09	7.64E-06	B9HEC0_POPTR	8.00E-12	Uncharacterized protein
CUFF.14284	−5.34	3.369	2.61E-09	8.76E-06	D7UCW0_VITVI	0	Laccase
B. Vaccinium arboreum							
CUFF.1549	2.29	3.15	1.31E-07	1.73E-03	A0A151STL0_CAJCA	3.00E-36	F-box/kelch-repeat protein At3g06240 family
CUFF.13406	−4.13	5.11	3.82E-07	2.35E-03	A0A103YHW1_CYNCS	1.00E-19	Stigma-specific protein Stig1
CUFF.34224	4.07	3.56	4.32E-07	2.35E-03	B9GWH1_POPTR	5.00E-41	Putative copper-transporting atpase 3 family protein
CUFF.4259	−2.50	5.45	4.47E-07	2.35E-03	A0A103XEL9_CYNCS	1.00E-28	Cupredoxin
CUFF.13402	−4.08	3.97	9.70E-07	2.68E-03	A0A103YHW1_CYNCS	7.00E-23	Stigma-specific protein Stig1
CUFF.17694	−2.90	4.42	9.18E-07	2.68E-03	A0A067JKU4_JATCU	4.00E-59	Laccase
CUFF.32389	2.51	3.31	6.37E-07	2.68E-03	A0A075EB50_9ERIC	0	EIN3-like protein EIL3
CUFF.57421	−2.37	2.43	1.02E-06	2.68E-03	I2BH27_LINUS	3.00E-39	Glycosyltransferase
CUFF.59288	2.21	5.45	8.89E-07	2.68E-03	E9LK45_VITVI	1.00E-50	Copper transporter
CUFF.61186	2.84	7.90	1.07E-06	2.68E-03	U5GPN1_POPTR	0.007	Uncharacterized protein
C. Contrasting response							
CUFF.2654	−6.55	5.56	4.63E-09	1.71E-04	A0A0B2Q009_GLYSO	3.00E-106	Prostaglandin G/H synthase 2
CUFF.24077	3.37	5.06	4.41E-08	8.13E-04	B9RDF5_RICCO	3.00E-155	Alcohol dehydrogenase, putative
CUFF.236	−4.02	4.42	1.08E-07	1.32E-03	A0A061DQL9_THECC	4.00E-37	Ralf-like 33, putative
CUFF.12615	−2.99	5.26	1.76E-07	1.35E-03	A0A061EMA1_THECC	8.00E-170	Leucine-rich repeat (LRR) family protein
CUFF.22809	−4.41	4.81	1.82E-07	1.35E-03	A0A118JUM8_CYNCS	8.00E-96	Cupin 1
CUFF.32460	−2.27	3.90	4.57E-07	1.57E-03	A0A022Q8S6_ERYGU	7.00E-39	40S ribosomal protein S21
CUFF.59789	−3.55	2.69	3.98E-07	1.57E-03	A0A061GSM4_THECC	8.00E-16	Uncharacterized protein
22424_g	−5.27	3.94	5.84E-07	1.80E-03	B9RTU6_RICCO	5.00E-12	Basic 7S globulin 2 small subunit
CUFF.59379	−4.42	4.00	6.57E-07	1.86E-03	A0A061GZR4_THECC	3.00E-121	Cytochrome P450 94A1
CUFF.10843	2.36	3.41	7.51E-07	1.91E-03	I6U574_9ROSA	8.00E-37	Dehydration-responsive element binding protein

Genes with differential expression in tetraploid VC (A) and diploid VA (B) blueberry species grown at pH 6.5 compared to growth at pH 4.5 in hydroponic medium, and differential response of VC compared to VA response (C). Genes are ordered by *p*-value in ascending order. logFC, log-abundance ratio as fold change; logCPM, log-average concentration in counts per million reads, normalized by library size; PValue, exact *p*-value for differential expression using the NB model; FDR, the *p*-value adjusted for multiple testing; uniprot_id, evalue and description belong to best matches against plant TrEMBL protein database. Genes without annotation are not shown here (for a full list of genes see Additional file 3: Table S1).

### Shared responses between VC and VA to growth at higher pH

Soil pH is a major component of soil chemistry. Many essential chemical elements exhibit high solubility in the soil matrix at near neutral pH. However, blueberries and their relatives are adapted to acidic soils [1–3, 62], where the availability of P, K, S, Ca, and Mg is reduced and the availability Al, Fe, Mn, Cu, Zn and B is enhanced [63]. Additionally, in acidic soils, the most abundant form of N is ammonium, whereas in neutral and basic soils the most abundant form of N is nitrate [63]. In the present experiment, both VA and VC exhibited transcriptional responses that highlight the relevance of Cu homeostasis in blueberries. VA and VC exhibited downregulation of a LPR1-like multicopper oxidase gene (gene.g23403.t1) whose product is involved in low P sensing in the root tip and interacts with the Cu uptake protein COPT2 [64]. Both species also upregulated a Cu transporter similar to heavy metal ATPase 5 (HMA5; CUFF.34224), a protein regulated by reactive oxygen species (ROS) and involved in either Cu extrusion or storage in other species [65]. The regulation of these activities suggests a response to Cu deficit that involves a reduction in Cu uptake and its storage in the vacuole. Such as soil particles, roots of plants grown in hydroponic systems have the capacity to adsorb metals in their cell walls in response to pH, with potential modification of their bioavailability. In this case, potential sequestration of soluble Cu by roots of blueberries grown at pH 6.5 may induce the response to low Cu, although further investigation is required for elucidation.

The plants also regulated detoxification processes in response to the modification in soil ion composition at higher pH. Thus, they upregulated the expression of a drug efflux transporter of the MATE family (CUFF.2426) similar to a member that improves tolerance to biotic stress in Arabidopsis [66]. In accordance to the decrease in H^+^ ions, these species exhibited downregulated transcription of a nitrate excretion transporter (CUFF.28540) that is coupled with H^+^ excretion activity in Arabidopsis grown at acidic pH [67]. Detoxification involving the accumulation of xenobiotics in the vacuole was also observed with the upregulation of genes encoding an ABC C family transporter (CUFF.30715) and glutathione S-transferase (CUFF.36740). ABC C family genes have been shown to transport glutathionated substrates across the tonoplast for sequestration or catabolism in Arabidopsis [68]. Additional ABC family transporters were either downregulated (CUFF.51372, CUFF.575), or upregulated (CUFF.8485). In other species, similar transporters have hypothesized or proven roles in external excretion and heavy metal tolerance [68].

Another focus in the transcriptional response to growth at higher pH in VA and VC is the cell wall. Primary cell walls are composed of cellulose microfibrils and structural proteins [69]. Secondary cell walls are less flexible than primary cell walls and contain lignin [70]. In this experiment, VA and VC exhibited limited DE of genes involved in primary cell wall deposition when grown at higher pH. VA and VC downregulated UDP-glucose dehydrogenase (CUFF.43587), which produces UDP-glucuronic acid, an important precursor in the synthesis of pectin and cellulose [71]. On the other hand, both VA and VC exhibited downregulation of the following genes involved in cell wall lignification when grown at higher pH. Lignin is a polymer of monolignols derived from phenylalanine through a multi-step process that involves several enzymes, being one of them hydroxycinnamoyl-CoA shikimate/quinate hydroxycinnamoyl transferase (HCT; CUFF.8465, [72]). Monolignols are glycosylated by glycosyltransferases (CUFF.57421) and transported to the apoplast by ABC B family transporters (CUFF.36985, [73]). In the Casparian strip, CASP-like proteins (CUFF.26419, CUFF.10512) recruit dirigent proteins (gene.g12238.t1) to regulate the localization of the lignin biosynthetic machinery [74]. Finally, monolignols are polymerized by specific isoforms of laccases (CUFF.14650, CUFF.37239) and peroxidases [75]. The pathway is partly regulated by the Mediator complex, composed of MED5a (CUFF.28551) and MED5b subunits, which is a transcriptional repressor of some lignin biosynthetic genes [76]. Overall, growth at higher pH of both VA and VC decreases the expression of multiple genes in the biosynthetic machinery of the lignin component of secondary cell walls. An enzyme involved in suberin biosynthesis (CUFF.17116) was also downregulated. Weakening of pectin cross-linkage by calcium ions was reported to lead to destabilization of the primary cell wall in acidic soil conditions [14], and in such case, modifications in the secondary cell wall may constitute an adaptive response to weakened primary cell walls in lignified root cells of blueberries grown at lower pH. Further investigation will be necessary to establish actual differences in cell wall content and composition and their putative role in adaptation to growth in acidic soils.

Higher pH also affected other physiological processes. For example, VC and VA shared downregulation of genes involved in the biosynthesis of signaling molecules in response to stress and salicylic acid (2OG oxygenase, CUFF.2391), ethylene (1-aminocyclopropane-1-carboxylate oxidase, CUFF.29933) or jasmonic acid (LOX1, CUFF.7443; LOX2, CUFF.23881; JMT, CUFF.48351). Upregulated metabolic enzymes also responding to ethylene were 2-oxoglutarate-dependent dioxygenases (CUFF.12564, CUFF.12562, CUFF.47236), which are related to iron-deficiency response in Arabidopsis [77]. Additionally, several transcription factors were either upregulated (CUFF.39185, CUFF.30535, CUFF.2863, and CUFF.14552) or downregulated (CUFF.21023, CUFF.21023, CUFF.39034, CUFF.1498) at higher pH. Enzymes involved in REDOX processes, such as cupredoxins (CUFF.4259, CUFF.4565), plantacyanin (CUFF.733), and cytochrome P450 (gene.g21243.t1, 43422_g) were also downregulated at higher pH. DE was observed in genes that encode kinases (CUFF.43578, CUFF.14188, CUFF.15544, and CUFF.40209) and stress-response proteins (CUFF.45828, CUFF.50959 and CUFF.24645). Interestingly, genes that affect cell growth were DE. VA and VC exhibited decreased transcription of phospholipid flippases similar to Arabidopsis genes encoding aminophospholipid ATPases (ALAs) 4/5, required for normal cell growth (CUFF.7136 and CUFF.7137), suggesting a mechanism that enhances cell division and elongation in the root tip under acidic pH, [78]. Furthermore, downregulation of a clathrin-binding protein located in endosomes and at the cell plate of dividing cells in Arabidopsis (CUFF.22673) suggest an alteration of root growth in *Vaccinium* spp. when exposed to higher pH, [79]. Finally, a gene that encodes a tonoplast-localized bidirectional sugar transporter (CUFF.30464) involved in the secretion of sugar in the rhizosphere [80] was also downregulated in VA and VC at higher pH.

Altogether, these results indicate the similar transcriptional responses of VA and VC to growth at pH 6.5, including detoxification mechanisms, modification of the cell wall lignin component and signaling pathways involved in cell growth. We discuss the contrasting transcriptional responses between these two species next.

### Contrasting responses between VC and VA to growth at higher pH

When visualized in an MDS plot, the global gene expression patterns of VC roots separated samples between pH 4.5 and pH 6.5 on the second dimension, while the samples of VA clustered together. Previous field observations suggested that VA exhibits tolerance to higher pH soils [7], but this had not been tested under controlled conditions. Additionally, previous research determined that VA exhibits deeper root systems than VC [7, 81], which might give VA access to deeper, more acidic soil strata. In the present study, the transcriptional response of VA to growth at pH 6.5 was not as pronounced as the transcriptional response of VC. These results, combined with the lack of rhizosphere acidification reported in VA [12], suggests that VA exhibits tolerance –rather than avoidance– to growth at higher soil pH. These findings resonate with field-trials where VC grafted onto VA rootstocks outperformed own-rooted VC in unamended soils [10]. To understand the mechanisms underlying the tolerance to pH in VA, several genes that showed DE in VA but not in VC will be discussed.

In this experiment, there were transcriptional responses related to nutrient uptake capacity. VA upregulated the expression of amino acid (CUFF.36901, [82]) and oligopeptide transporters (CUFF.51980, [83]) with functions in uptake of organic N sources in Arabidopsis. Considering that uptake of organic N by *Vaccinium* spp. has been previously documented [84], these findings suggest that in higher pH soils, where ammonium bioavailabilty is low, VA might resort to organic N sources for its nutrition. VA also regulated a number of genes potentially involved in copper trafficking [65], with upregulation of the metal-chelate transporter YSL3 (CUFF.41504) and Cu uptake (COPT5, CUFF.59288) and mobilization (COPT1, CUFF.47667) transporters, and downregulation of the Cu efflux transporter HMA5 (CUFF.45778). Additionally, a ZIP1-like Zn transporter involved in the response to Zn deficiency [85] was upregulated (CUFF.17984). These results suggest an increase by VA in uptake and homeostasis of divalent metals, which are less bioavailable at higher pH, with emphasis on micronutrients Cu and Zn. Additional DE of nutrient-related genes was focused on limiting the uptake or enhancing the compartmentalization of minerals that are readily available at higher pH. For example, genes that encode K (CUFF.45187) and Mg (CUFF.36078) uptake transporters were downregulated and a gene that encodes Ca transport into the vacuole was upregulated (CUFF.32105) in VA. Finally, an aquaporin involved in water and ion transport was downregulated (CUFF.8836). Altogether, these findings reveal some of the genes whose regulation contribute to balance nutrient uptake and homeostasis in relation to an effective response for VA tolerance to growth at higher pH.

Further downregulation was observed in genes related to the primary and secondary cell wall construction and repair in VA grown at higher pH, in addition to those previously described. For example, VA downregulated a boron efflux transporter (CUFF.48824), required for crosslinking of the pectic polysaccharide rhamnogalacturonan II (RG-II), as well as a xylanase (CUFF.51472) and a bifunctional α-L-arabinofuranosidase/β-D-xylosidase (CUFF.53455), wall-modifying enzymes that act on hemicellulose at the cell wall. Finally, genes that encode proteins involved in monolignol production (CUFF.12829) and polymerization (LAC7, CUFF.9553), and in suberin biosynthesis (gene.g8711.t1), were also downregulated. The modifications performed by VA to the cell wall machinery are less drastic in comparison to VC in view of the greater number of genes that VC regulates and VA doesn’t, coding proteins that modulate cell wall extensibility (expansins, xyloglucan endotransglucosylase/hydrolases and endo-1,4-beta-D-glucanases), cell wall plasticity (pectinesterases, polygalacturonases, pectate lyases and pectin acetylesterases; [86]) and lignin biosynthesis (peroxidases), most of which are downregulated (Table A in Additional file 3: Table S1). Previously, changes in cell wall composition have been observed in the cell walls of plants under abiotic stress, which can be concomitant with low pH conditions [86].

A small set of transcription factors were specifically DE in the roots of VA in comparison to VC. Some downregulated genes were involved in producing the Casparian band (MYB36-like, CUFF.11761), cell wall formation (CUFF.1106) and ROS-mediated responses (CUFF.1500, CUFF.39809, CUFF.1501), while upregulated genes included a negative regulator of resistance genes (WRKY48-like, CUFF.3660), an ethylene signaling mediator (EIN3-like protein EIL3, CUFF.32389) and a calmodulin-binding transcription activator (CUFF.56809). Additional transcription factors that were DE involved organ development (CUFF.8326, CUFF.22347) and stress response (CUFF.4666, CUFF.38889, gene.g3093.t1, CUFF.53346, CUFF.751). Downregulation of a negative regulator of defense response with MYB30 ubiquitination activity (CUFF.39430) was also observed. Interestingly, different genes that encode homologs to Arabidopsis *Sensitive to Proton Rhizotoxicity 1* (*STOP1*) were downregulated in VA (CUFF.54376) and VC (CUFF.15468 and CUFF.29512). Previous research showed that *STOP1* is upregulated under low pH stress and participates in H^+^ and Al tolerance [87]. *STOP1* has also been associated with mechanisms for stabilizing the cell wall [87]. These functions are consistent with *STOP1*, an H^+^ efflux transporter and multiple genes targeting the cell wall being downregulated at higher pH in both species. Intriguingly, while Al-responsive genes were not DE in VA, Al-activated malate transporters (CUFF.11109 and CUFF.20376) were upregulated in VC at higher pH. The pleiotropic effects of *STOP1* suggest the activation of different responses in VA and VC by their respective genes that affect their tolerance capacity to higher pH. In addition to RNA-Seq, further validation by gene sequencing and qPCR will provide additional resources in future experiments to elucidate the role of *STOP1*.

DE of genes involved in hormone-mediated responses was also observed at higher pH in VA. Upregulated genes were involved in biosynthesis of ethylene (CUFF.25643, CUFF.14868, CUFF.22603) and jasmonic acid (89766_g, CUFF.15527) and also gibberellin deactivation (CUFF.45537), whereas downregulated genes were involved in brassinosteroid biosynthesis (CUFF.18539), jasmonic acid metabolism (CUFF.14932) and cytokinin deactivation (CUFF.39626). Also, three hormone transporters of the ABC family were downregulated (CUFF.15221, CUFF.15223, CUFF.42795). Not only signaling was mediated through hormones, but also genes involved in Ca^2+^-dependent signaling were DE (CUFF.40710, CUFF.9375). Signaling cascades induce transcriptional changes that affect a vast number of physiological processes, some of which were already discussed. Additionally, DE is observed in genes associated with senescence (CUFF.17945, CUFF.17949, CUFF.1386), detoxification (Glutathione S-transferases [GSTs], 75717_g and CUFF.30506; heavy metal transport/detoxification protein, CUFF.16889), components for endosomal trafficking (CUFF.12767, gene.g4545.t1, CUFF.49167, CUFF.17075), carbon transport (CUFF.51281) and lipid transport (CUFF.44077, CUFF.18533, CUFF.18546).

Finally, a few genes related to disease resistance were either upregulated (CUFF.1549, CUFF.29893, 78263_g, CUFF.18079, CUFF.20096) or downregulated (CUFF.54434, CUFF.8327, CUFF.2543) in VA, in contrast with the response in VC that involved a much larger number of genes (Table A in Additional file 3: Table S1). Interestingly, despite the vast defense response, VC exhibits significant downregulation of a gene involved in the production of oxylipins (CUFF.2654) in comparison to VA (Table 1c). In response to the changes in both biotic and oxidative stresses, VA regulated genes involved in the secondary metabolism implied in isoprenoid (CUFF.42000, gene.g11090.t1, gene.g19172.t1, CUFF.31569), flavonoid (CUFF.20951, CUFF.34078, CUFF.51711) and sterol (CUFF.57550) biosynthesis. While previous studies have found extensive crosstalk between biotic and abiotic stress response transcriptional networks [88], disease might be a factor affecting the growth of *Vaccinium* spp. at higher pH. In this scenario, VA and VC exhibit large transcriptional differences in relation to the cell wall and secondary metabolism, which participate in the mechanical and chemical protection of cells. The less extensive response to disease and healthy growth of VA at higher pH would be an indication of a better protection against pathogens than VC. Moreover, transcriptional responses to disease pressure might also play a role in the dissimilar soil adaptations of these species.

## Conclusions

The present study compared the root transcriptomes of VA and VC rooted cuttings that were grown hydroponically in either a preferred low (pH 4.5) or stressful higher pH (pH 6.5). While both VA and VC exhibited DE of nutrition- and detoxification-related genes in response to growth at higher pH, VA exhibited a wider transcriptional response towards Cu homeostasis suggesting a response to Cu scarcity. Both VA and VC exhibited downregulation of multiple genes involved in primary and secondary cell wall biosynthetic machinery at higher pH. However, the additional changes triggered by VC suggest a reduced adaptability of the cell wall in comparison to VA. Moreover, VA and VC downregulated different genes similar to the transcription factor *STOP1*, which is involved in the response to acidic soils in other species, suggesting variation in their respective responses. Altogether, our findings suggest that VA adaptation to high pH soil is the product of an effective transcriptional regulation primarily modifying its nutrition, detoxification and cell wall gene networks.

## Additional files

Additional file 1: Figure S1. Hydroponic setup. (JPEG 2413 kb)

Additional file 2: Figure S2. MA-plot of normalized counts. (TIFF 710 kb)

Additional file 3: Table S1. Lists of genes with differential expression in blueberry under pH stress. Significant genes in VC (A), VA (B) and their different response (C). Based on the quasi-likelihood F test. *p* < 0.05 after Benjamini & Hochberg false discovery rate (FDR) correction. (XLSX 1020 kb)

Additional file 4: Table S2. Sequence similarity-based annotation of blueberry gene models. Annotations obtained from proteins in the TrEMBL database for plants and *Arabidopsis thaliana* sequences in NCBI. *Arabidopsis* descriptions include, when available, enzyme and pathway information from KEGG and AraCyc [90]. (TXT 7236 kb)

Additional file 5: Table S3. Enrichment analysis of GO terms. Genes showing differential expression in VA (A) and VC (B) were subjected to singular enrichment analysis in AgriGO. The table contains output from the analysis performed using the combination AgriGO and REVIGO tools. GO terms with FDR < 0.5 after Yekutieli multi-test in AgriGO were used on REVIGO and searched for genes with that specific annotation. (XLSX 184 kb)

Additional file 6: Table S4. Summary of discussed genes. Collection of genes present in discussion organized by function. The columns represent category/gene ID, description of best UniProt protein homolog, description of best *Arabidopsis* protein homolog, log fold change (FC) in *V. arboreum* and logFC in *V. corymbosum*. (XLSX 17 kb)

## Abbreviations

DE: Differential expression/differentially expressed
FCR: Ferric chelate reductase activity
FDR: False discovery rate
GO: Gene Ontology
MDS: Multidimensional scaling
VA: *Vaccinium arboreum*
VC: *Vaccinium corymbosum*
