# Supplementary figures and images for: Regulation of gene expression in roots of the pH-sensitive Vaccinium corymbosum and the pH-tolerant Vaccinium arboreum in response to near neutral pH stress using RNA-Seq
Source: BMC Genomics. 2017 Aug 7;18:580. doi: 10.1186/s12864-017-3967-0 (PMC5547544; doi:10.1186/s12864-017-3967-0)

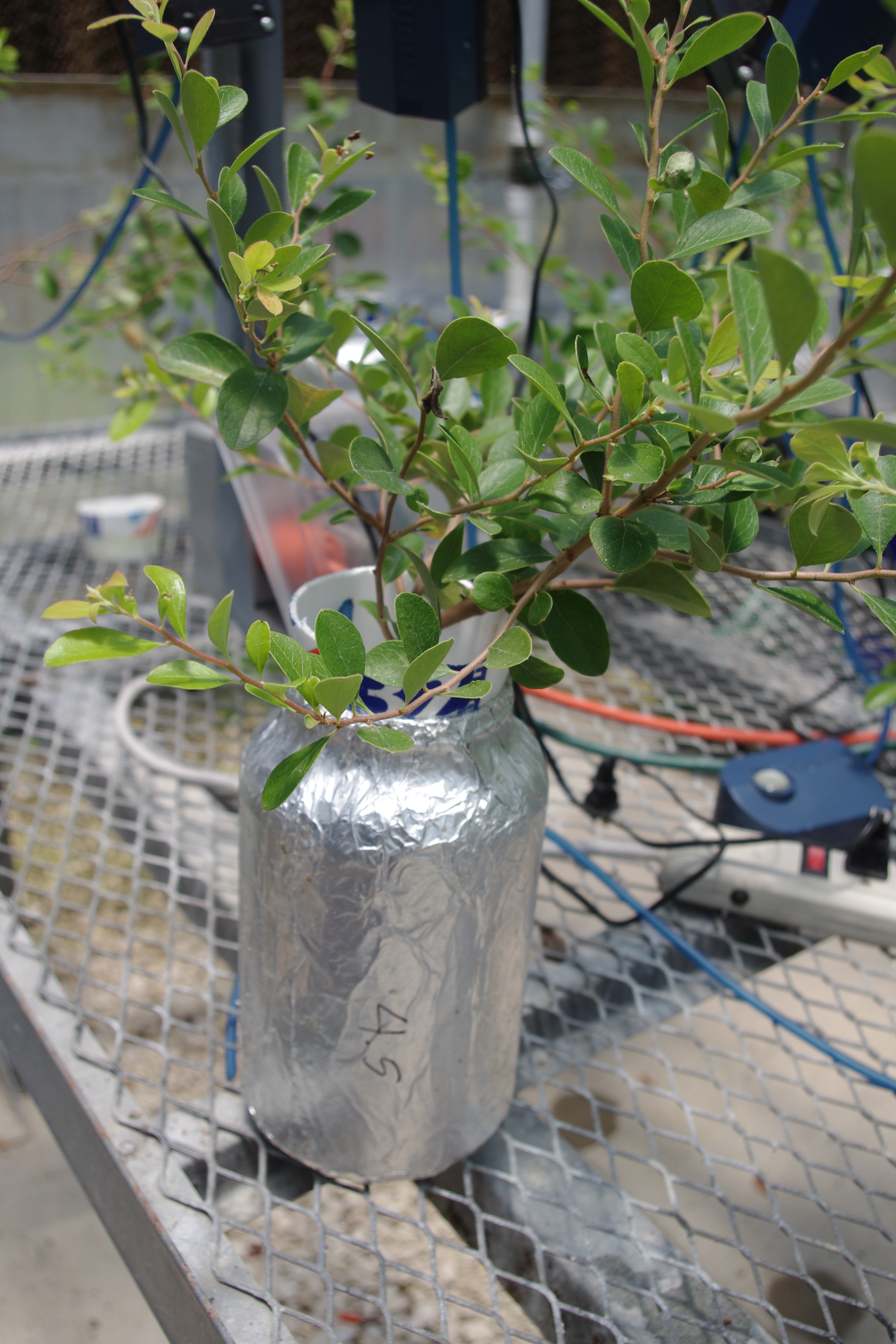

Supplement: Supplementary file 1 — Hydroponic setup. (JPEG 2413 kb) [file 12864_2017_3967_MOESM1_ESM.jpg]

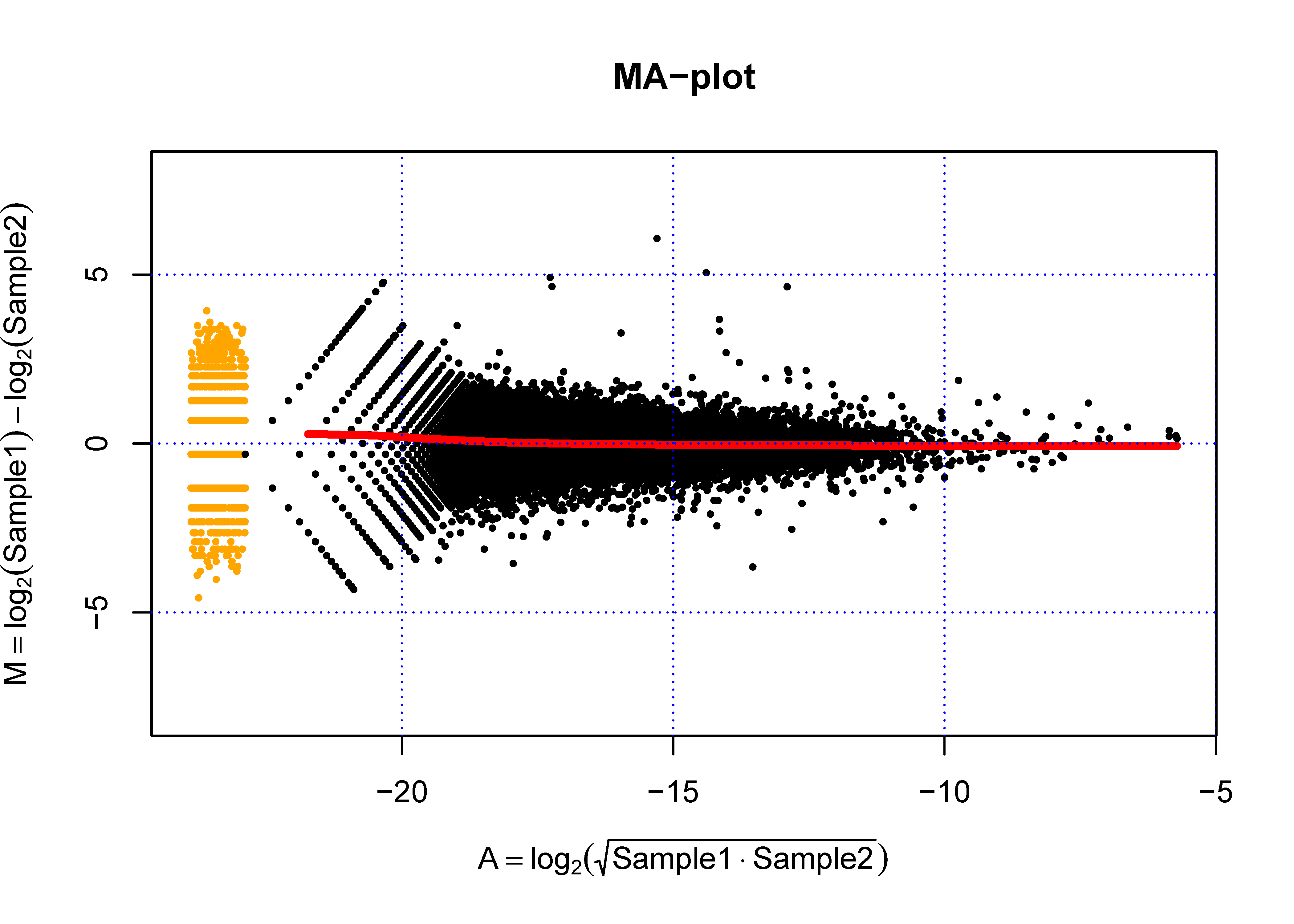

Supplement: Supplementary file 2 — MA-plot of normalized counts. (TIFF 710 kb) [file 12864_2017_3967_MOESM2_ESM.tiff]
